# Domestication Origin and Breeding History of the Tea Plant (*Camellia sinensis*) in China and India Based on Nuclear Microsatellites and cpDNA Sequence Data

**DOI:** 10.3389/fpls.2017.02270

**Published:** 2018-01-25

**Authors:** Muditha K. Meegahakumbura, Moses C. Wambulwa, Miao-Miao Li, Kishore K. Thapa, Yong-Shuai Sun, Michael Möller, Jian-Chu Xu, Jun-Bo Yang, Jie Liu, Ben-Ying Liu, De-Zhu Li, Lian-Ming Gao

**Affiliations:** ^1^Key Laboratory for Plant Diversity and Biogeography of East Asia, Kunming Institute of Botany, Chinese Academy of Sciences, Kunming, China; ^2^Germplasm Bank of Wild Species in Southwest China, Kunming Institute of Botany, Chinese Academy of Sciences, Kunming, China; ^3^College of Life Science, University of Chinese Academy of Sciences, Kunming, China; ^4^Genetics and Plant Breeding Division, Coconut Research Institute of Sri Lanka, Lunuwila, Sri Lanka; ^5^Biochemistry Department, South Eastern Kenya University, Kitui, Kenya; ^6^Department of Botany, Dinhata College, Dinhata, India; ^7^Key Laboratory of Tropical Forest Ecology, Xishuangbanna Tropical Botanical Garden, Chinese Academy of Sciences, Mengla, China; ^8^Royal Botanic Garden Edinburgh, Edinburgh, United Kingdom; ^9^Centre for Mountain Ecosystem Studies, Kunming Institute of Botany, Chinese Academy of Sciences, Kunming, China; ^10^Tea Research Institute of Yunnan Academy of Agricultural Sciences, Menghai, China

**Keywords:** *Camellia sinensis*, nSSR, cpDNA sequence, domestication center, breeding history

## Abstract

Although China and India are the two largest tea-producing countries, the domestication origin and breeding history of the tea plant in these two countries remain unclear. Our previous study suggested that the tea plant includes three distinct lineages (China type tea, Chinese Assam type tea and Indian Assam type tea), which were independently domesticated in China and India, respectively. To determine the origin and historical timeline of tea domestication in these two countries we used a combination of 23 nSSRs (402 samples) and three cpDNA regions (101 samples) to genotype domesticated tea plants and its wild relative. Based on a combination of demographic modeling, NewHybrids and Neighbour joining tree analyses, three independent domestication centers were found. In addition, two origins of Chinese Assam type tea were detected: Southern and Western Yunnan of China. Results from demographic modeling suggested that China type tea and Assam type tea first diverged 22,000 year ago during the last glacial maximum and subsequently split into the Chinese Assam type tea and Indian Assam type tea lineages 2770 year ago, corresponding well with the early record of tea usage in Yunnan, China. Furthermore, we found that the three tea types underwent different breeding histories where hybridization appears to have been the most important approach for tea cultivar breeding and improvements: a high proportion of the hybrid lineages were found to be F_2_ and BCs. Collectively, our results underscore the necessity for the conservation of Chinese Assam type tea germplasm and landraces as a valuable resource for future tea breeding.

## Introduction

The tea plant, *Camellia sinensis* (L.) O. Kuntze, is an important economic tree crop (Mondal et al., 2004; Chen and Chen, 2012) that is currently grown in over 52 countries; China and India are the two largest global tea producers (FAOSTAT, 2015). Its taxonomic classification, however, is still a subject of great debate due, in part, to the plasticity of morphological traits that are used to discern tea taxa (Sealy, 1958; Wight, 1962; Banerjee, 1992; Ming, 2000). According to the classification system of Ming (2000), the cultivated tea plant is currently treated as two varieties, i.e., *C. sinensis* var. *sinensis* (China type tea) and *C. sinensis* var. *assamica* (Masters) Chang (Assam type tea). Although the Cambod type tea (*C. assamica* subsp. *lasiocalyx* Planch) has been treated as a synonym of *C. sinensis* var. *assamica*, recent genetic analyses have revealed that the Cambod type tea is in fact a hybrid between the China and Assam type teas (Wambulwa et al., 2016a). In addition, *C. sinensis* var. *assamica* in China and India has been shown to belong to two distinct genetic entities (Meegahakumbura et al., 2016). Apart from *C. sinensis*, there are eleven more species of *Camellia* sect. *Thea* that occurred in China and several species were used as beverage “tea” in Yunnan, Southwest China (Ming, 2000; Ming and Bartholomew, 2007). *Camellia taliensis* is a close wild relative of domesticated tea that is proposed to have been involved in the domestication and breeding of *C. sinensis* var. *assamica* in Yunnan, China (Li et al., 2015). A comprehensive genetic definition of tea lineages may help to resolve taxonomic delineations among tea taxa that are currently domesticated in China.

Crop domestication is a dynamic and ongoing process where wild progenitors give rise to landraces, which in turn, form the genetic basis of modern cultivars (Diamond, 1997; Zohary, 1999). Domesticated over 3,000 years ago, tea is one of the earliest tree crop species in China (Yamanishi, 1995). Recent excavations of the “Han Yangling Mausoleum” in Xi'an, Shaanxi province, China, revealed that tea drinking was popular among emperors in the Han dynasty more than 2,100 years ago (Lu et al., 2016) providing strong evidence that China type tea originated in China and has a long history of utilization and cultivation in the region. The origin of domestication of the tea plant in China, however, has long been controversial. Several regions, such as Sichuan and Yunnan provinces of China, Assam of India, and Indo-Burma, have long been suspected to be the origin of tea domestication (Stuart, 1919; Wight, 1959; Lu, 1974). In a recent study based on nuclear microsatellite (nSSR) data, two domestication events of tea in China and one in India have been detected (Meegahakumbura et al., 2016). However, the patterns of divergence among these three tea lineages as well as the historical timelines associated with their origins of domestication remain to be explored.

Tea breeding has a long history in China, and can be traced back over 1,000 years (Wu, 1987). Historically, cultivars were grown and selected from naturally occurring seed sources (Chen et al., 2007) and subsequently propagated vegetatively (Yao and Chen, 2012). More recently, artificial pollination and hybridization among selected tea types or with wild relatives have become an important breeding strategy for modern tea cultivar development (Wachira et al., 1997; Sharma et al., 2010). To date, over 5,100 accessions of tea germplasm have been selected and conserved in China and India (Chen et al., 2007; Das et al., 2012; Yao and Chen, 2012) and all genetic stocks for most tea growing countries were directly or indirectly introduced from either China or India (Meegahakumbura et al., 2016), although some secondary exchange may have occurred between tea producing nations (Gunasekare, 2012; Sriyadi et al., 2012; Wambulwa et al., 2017). Unfortunately, the breeding history of tea germplasm and the extent of exchange of tea genetic resources between China and India remains poorly understood. Because the morphological characters that are traditionally used to define cultivars are highly plastic and easily influenced by environmental conditions (Yao et al., 2008), a molecular analysis that can not only discern patterns of lineage but also the origin of tea germplasm is required.

To further elucidate the origin of the domestication and breeding history of the tea plant in China, we used a combination of nSSR (402 samples) and cpDNA (101 samples) markers to genotype samples of tea landraces, cultivars and their wild relatives collected from China and India. nSSRs, which are co-dominant markers and bi-parentally inherited (Powell et al., 1996) were incorporated into demographic models and NewHybrids analyses were carried out to investigate the domestication history (Carnille et al., 2012; Diez et al., 2015). cpDNA, which is maternally inherited in *Camellia* (Kaundun and Matsumoto, 2011), was used to generate network and neighbor joining (NJ) trees to study the origins of maternal lineages associated with tea domestication. Specifically, we aimed to (1) investigate the demographic history and domestication origin of the tea plant, (2) explore patterns of tea breeding history in the domestication centers of China and India, and (3) clarify the genetic relationships, and origins of tea cultivars in both countries. Our findings will contribute directly to the development of modern tea breeding programmes and future tea germplasm conservation strategies.

## Materials and methods

### Sampling

A total of 402 samples from China and India were included in the present study, 392 samples were from a *Camellia sinensis* dataset of a previous study (Meegahakumbura et al., 2016) and 10 samples came from the closely related wild relative, *C. taliensis* (Table S1). Based on our previous work involving the 392 tea samples, 276 samples were grouped as “pure” tea types and the remaining 116 were classified as a Mosaic group representing hybrids (Meegahakumbura et al., 2016; Table S2). The tea samples were divided into landraces (samples collected from ancient tea trees improved by traditional selection methods) and cultivars (samples generated from modern tea breeding programmes). Hereafter, *C. sinensis* var. *sinensis* will be referred to as “China type tea”; *C. sinensis* var. *assamica* occurring in China as “Chinese Assam type tea”; and *C. sinensis* var. *assamica* that occurs in India as “Indian Assam type tea.” All Cambod tea accessions were treated as mosaic cultivars based on our previous study (Meegahakumbura et al., 2016). Finally, a total of 12 subgroups were defined for the convenience of presentation and discussion. These include: CT: *C. taliensis*; CCL: Landrace of China type tea in China; CCC: Cultivars of China type tea in China; CCCM: Mosaic cultivars of China type tea in China; CIC: Cultivars of China type tea in India; CICM: Mosaics cultivars of China type tea in India; ACL: Landraces of Chinese Assam type tea; ACLM: Mosaic landraces of Chinese Assam type tea; ACC: Cultivars of Chinese Assam type tea; ACCM: Mosaic cultivars of Chinese Assam type tea; AIC: Cultivars of Indian Assam type tea; AICM: Mosaic cultivars of Indian Assam type tea. Vouchers for most of the samples were collected and deposited in the herbarium of Kunming Institute of Botany, Chinese Academy of Sciences (KUN).

### DNA extraction, nSSR genotyping, and cpDNA sequencing

Extraction of total genomic DNA was carried out using a CTAB method with slight modifications (Doyle and Doyle, 1990). For the 10 samples of *C. taliensis*, nSSR genotyping was conducted using a set of 23 nSSR primers as described in Meegahakumbura et al. (2016). A total of 101 samples were selected to represent all nSSR NJ tree clades, tea types and origins that were found in our previous study (Meegahakumbura et al., 2016). These samples were then sequenced for three cpDNA intergenic spacer regions: *ndh*F-*rpl*32, *trn*SGG-*trn*Sr, and *trn*Sf1-*trn*GGG (Yang et al., 2013). Detailed information of the primer sequences and the methodology for cpDNA sequencing are given in our recent study (Wambulwa et al., 2016b). The sequences of the 31 haplotypes were deposited in GenBank (Table S3).

### nSSR data analysis

A total of 23 nSSR loci were used to genotype all 402 samples (Table S1), and the nSSR data were checked manually as described in Meegahakumbura et al. (2016). Using the admixture model in STRUCTURE (Prichard et al., 2000), a population structure analysis was performed for samples grouped as “pure” and Mosaic. These were evaluated for 1–10 genetic clusters (*K*) with 20 permutations for each *K* value. STRUCTURE was run with 100,000 generations of burn-in followed by 100,000 Markov Chain Monte Carlo (MCMC) iterations. To estimate the optimal number of genetic clusters, Δ*K* was obtained using the method of Evanno et al. (2005) as implemented in STRUCTURE HARVESTER web v.0.6.94 (Earl and von Holdt, 2012). The optimal *K* value based on the Log-likelihood method was calculated with STRUCTURE HARVESTER. Genetic diversity indices were then calculated for the grouped dataset (pure tea types) excluding the Mosaic samples. The observed heterozygosity (*Ho*) and percentage of rare alleles (*R*_*A*_%) (with frequencies ≤0.05 according to White et al., 1999) for each group were calculated in GenA1Ex v.6.5b4 (Peakall and Smouse, 2006). The total allele number (*A*), gene diversity (*Hs*), allele richness (*A*r) and inbreeding coefficient (*F*is) for each tea group were estimated with FSTAT v.2.9.3.2 (Goudet, 1995).

**Figure 1:**
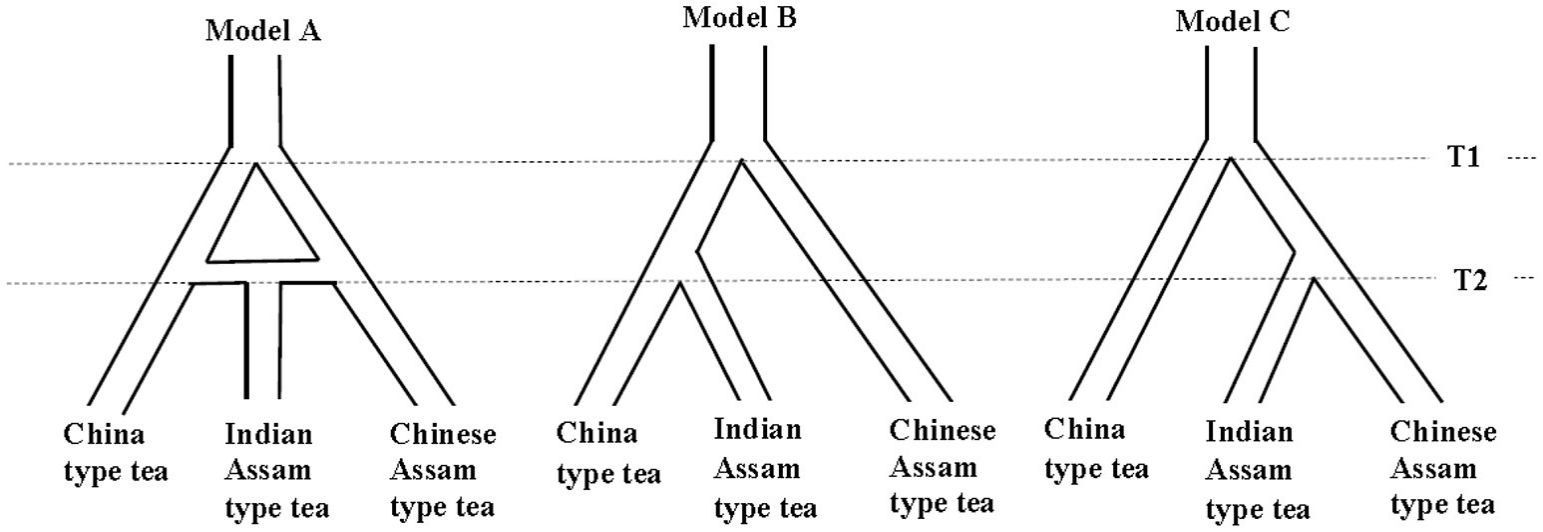
Three demographic history models tested for tea domestication.

We computed three hypothetical models (Figure 1) to infer the possible demographic history and genetic divergence of the tea plant in China and India. “Model A” assumed that Indian Assam type tea originated via hybridization between China type tea and Chinese Assam type tea. In “Model B,” Indian Assam type tea was assumed to have diverged from China type tea, while “Model C” assumed Indian Assam type tea derived from Chinese Assam type tea. A stepwise nSSR allele evaluation method was used in this analysis. The grouped nSSR dataset of 276 samples, excluding samples categorized as Mosaics, was used to generate 11 summary statistical parameters in ARLEQUIN v.3.5 (Exocoffier and Lischer, 2010), following the method of Sun et al. (2014). A standard algorithm of the Approximate Bayesian Computing (ABC) toolbox (Wegmann et al., 2010) and the computer program Fastsimcoal (Exocoffier and Foll, 2011) was employed. A total of 3 × 10^6^ simulated samples (1 × 10^6^ for each model) were generated to compare the three models. Based on these simulations, a “Bayes-factor” (BF) was calculated to identify the best-fit model for the dataset. The ABC toolbox was also used to calculate the mutation rate for each of the 23 nSSR loci where the mean mutation rate was used to estimate the divergence time. To decrease redundancies associated with the summary statistics, partial least square (PLS) components for a total of 11 statistics were recalculated using the R script of the ABC toolbox (Wegmann et al., 2010). Following the root mean square error plot and conversion equations inferred for 10,000 samples simulated by the standard algorithm, a total of 6 PLS components were used to analyse each of the three models. A regression-adjusted general linear model (GLM) was employed to generate posterior probability distributions for each parameter and to calculate the divergence time between each genetic lineage assumed in the models.

A NewHybrids analysis (Anderson and Thompson, 2002), was carried out following Wambulwa et al. (2016a), with slight modifications. Based on the results of a previous study (Meegahakumbura et al., 2016), three distinct genetic groups were used as parents in the NewHybrids analysis for different datasets: China type tea (*C. sinensis* var. *sinensis*), Chinese Assam type tea (*C. sinensis* var. *assamica* in China), and Indian Assam type tea (*C. sinensis* var. *assamica* in India). For the dataset composed of tea samples collected from India, China type tea and Indian Assam type tea were assigned as parents given that Chinese Assam type tea from India has not been reported (Meegahakumbura et al., 2016). For the dataset of China type tea samples from China (CCCM), China type tea and Chinese Assam type tea were used as parents. In addition, for the dataset of Chinese Assam samples from Yunnan Province of China (ACLM+ACCM), Chinese Assam type tea and *C. taliensis* were used as parents, because *C. taliensis* naturally occurs in sympatric populations with Chinese Assam type tea and has been recognized as contributing to the domestication of Assam type tea in China (Li et al., 2015).

### cpDNA sequence data analysis

The original trace files of the sequences for each region were assembled and edited using Sequencher v.5.0 (Genecodes Co., Michigan, USA). The final sequences of the three cpDNA were concatenated using SequenceMatrix v.1.7.8 (Vaidya et al., 2011) and then aligned in Geneious Pro v.4.8.5 (Biomatters Ltd., USA), and subsequently, manually adjusted where necessary. The combined sequences were assigned to different haplotypes using DNASP v.5.10 (Librado and Rozas, 2009), with each indel treated as a single mutation event. A neighbor joining (NJ) tree was generated in MEGA v.6 (Tamura et al., 2013) with the pairwise deletion and K2P distance model. A median joining haplotype network was constructed using the program NETWORK v.4.6.1.3 (available at http://www.fluxus-engineering.com) with the maximum parsimony (MP) criterion.

## Results

### Genetic diversity

The summary statistics for the different genetic groups is given in Table 1. The highest genetic diversity (*Hs* = 0.731, *Ho* = 0.690) was observed for AIC, followed by CIC (*Hs* = 0.706, *Ho* = 0.652), while *C. taliensis* (CT) had the lowest genetic diversity (*Hs* = 0.642, *Ho* = 0.517). As expected, landraces of both China type tea in China (CCL) and Chinese Assam type tea (ACL) exhibited relatively higher gene diversity (0.702 and 0.642) than those of cultivars (0.684 and 0.625). The highest allelic richness (*Ar*) was recorded for CCL and AIC (4.9), while the lowest was for ACC (4.2). The highest number of alleles (A) was observed in CCC (225), followed by AIC (200), and the lowest was recorded for CIC (102). ACL had the highest percentage of rare alleles (*R*_*A*_: 48.62%), followed by ACC (47.16%), while no rare alleles were found in CIC.

**Table 1:** Genetic diversity parameters of the grouped dataset of landraces and modern cultivars excluding the mosaic group.

**Group**	***N***	***A***	***R_*A*_*%**	***Ar***	***Ho***	***Hs***	***F*is**
CT	10	120	31.67	4.3	0.517	0.624	0.171
CCL	36	189	37.57	4.9	0.679	0.702	0.034
CCC	89	225	45.78	4.8	0.620	0.684	0.094
CIC	6	102	0	4.4	0.652	0.706	0.076
ACL	45	181	48.62	4.3	0.547	0.642	0.147
ACC	51	176	47.16	4.2	0.548	0.625	0.122
AIC	46	200	38.50	4.9	0.690	0.731	0.055
Total	283	301		5.6	0.608	0.673	0.1

*N, number of samples; A, number of alleles; R_A_%, Percentage of rare alleles; Ho, observed heterozygosity; He, gene diversity; Fis, inbreeding coefficient. CT, Camellia taliensis; CCL, Landrace of China type tea in China; CCC, Cultivars of China type tea in China; CIC, Cultivars of China type tea in India; ACL, Landraces of Chinese Assam type tea; ACC, Cultivars of Chinese Assam type tea; AIC, Cultivars of Indian Assam type tea*.

### Structure analysis

**Figure 2:**
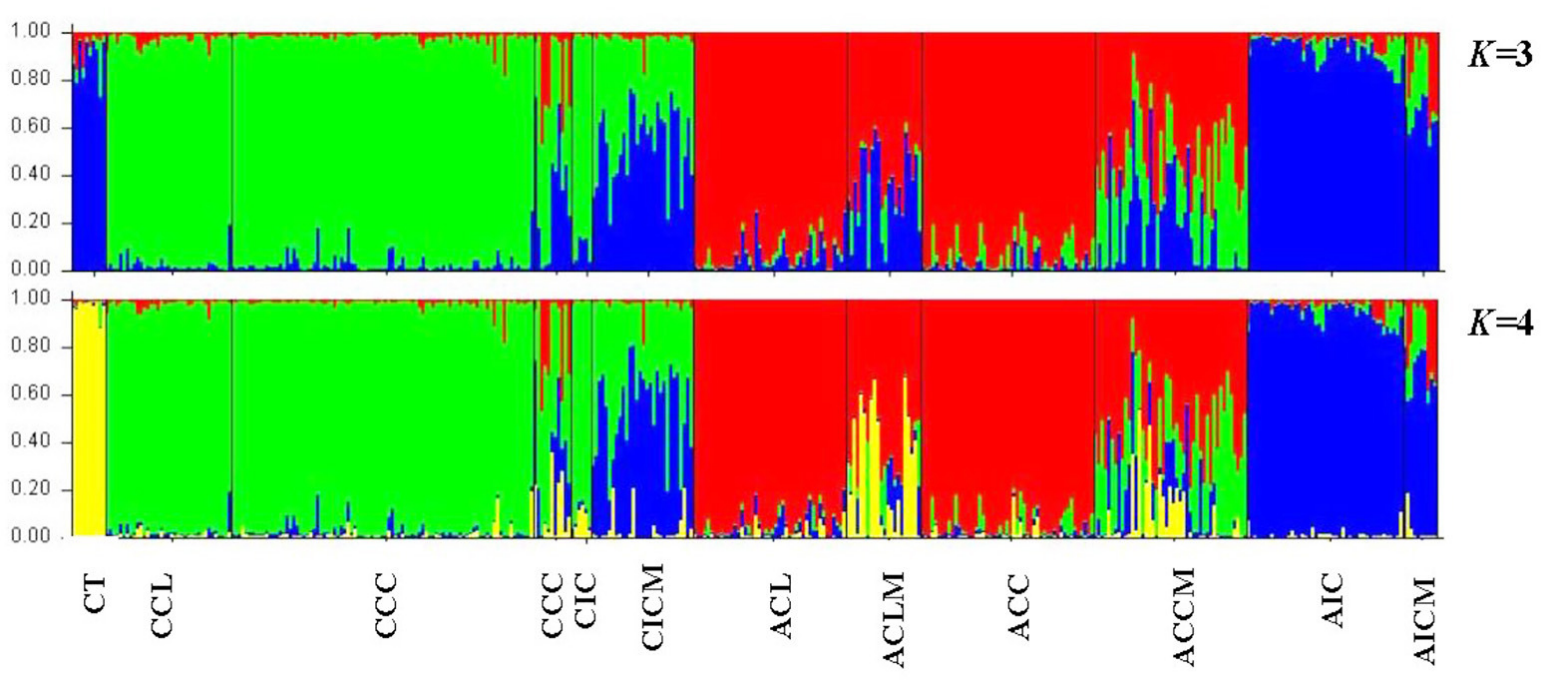
Results of the STRUCTURE analysis at *K* = 3 and 4 based on nSSR data for a total of 402 tea samples collected from China and India assigned according to their groups and respective mosaic groups.

The optimal *K* value was found to be two and four based on the Δ*K* (Figure S1) and the Log-likelihood method (Figure S2), respectively. The STRUCTURE results with the “admixture model” are given with *K* = 3 and 4, but not for *K* = 2 because three distinct tea types were defined in our previous study (Meegahakumbura et al., 2016; Figure 2). When *K* was 3, three genetic groups were found, representing the China type tea group (green), Chinese Assam type tea (red), and Indian Assam type tea + *C. taliensis* (blue) (Figure 2). Interestingly, Indian Assam type tea was not separated from *C. taliensis* at *K* = 3. With *K* = 4, *C. taliensis* was separated from Indian Assam type tea and formed a distinct genetic group (yellow) (Figure 2). Samples of the Mosaic group showed genetic admixtures between tea types or between tea types and *C. taliensis*. For example, ACCM samples showed admixture between China type tea, Chinese Assam type tea and *C. taliensis*; CICM samples showed admixture between China type tea and Indian Assam type tea; and AICM samples showed admixture between Indian Assam type tea and China type tea. In most accessions, ACLM showed a mixed genetic make-up between Chinese Assam type tea and *C. taliensis*. Compared to a previous study by Meegahakumbura et al. (2016), three samples (LMM159, LMM337, and LMM649) of ACL were re-assigned to the Mosaic group (ACLM) with a genetic admixture between Chinese Assam type tea and *C. taliensis*. Overall, a total of 119 samples were assigned to the Mosaic (hybrids) group in the present study.

### Demographic modeling analysis

The mean mutation rate (μ) across the 23 nSSR loci was estimated to be 0.515 × 10^−3^ which was used to evaluate the three models described above. The highest Bayes factor support (BF = 75.3) was found for Model C which assumed that the Indian Assam type tea and Chinese Assam type tea lineages diverged at the time T2 (Figure 1), suggesting this to be the best-fit model for the demographic history of tea domestication. Model A (the hybrid model) had the lowest Bayes factor support (BF = 0.0477), and Model B with the assumption of Indian Assam type tea and China type tea diverging at time T2 also exhibited a low Bayes factor support (BF = 1). Therefore, Model C was used to estimate population divergent times T1 and T2. The divergence time between China type tea and Assam tea (T1) was estimated with 1,875 generations, and the divergence time between Indian Assam type tea and Chinese Assam type tea (T2) was estimated with 231 generations. With an estimated generation time for the tea plant of around 12 years (Duke, 1983), we calculated that China type tea and Assam tea diverged approximately 22,552 years ago (95% Highest Probability Density Interval [HPDI]: 2,598–1,043,593), while Chinese and Indian Assam type tea diverged more recently, approximately 2,770 years ago (95% HPDI: 1,200–235,932).

### Newhybrids analysis

**Figure 3:**
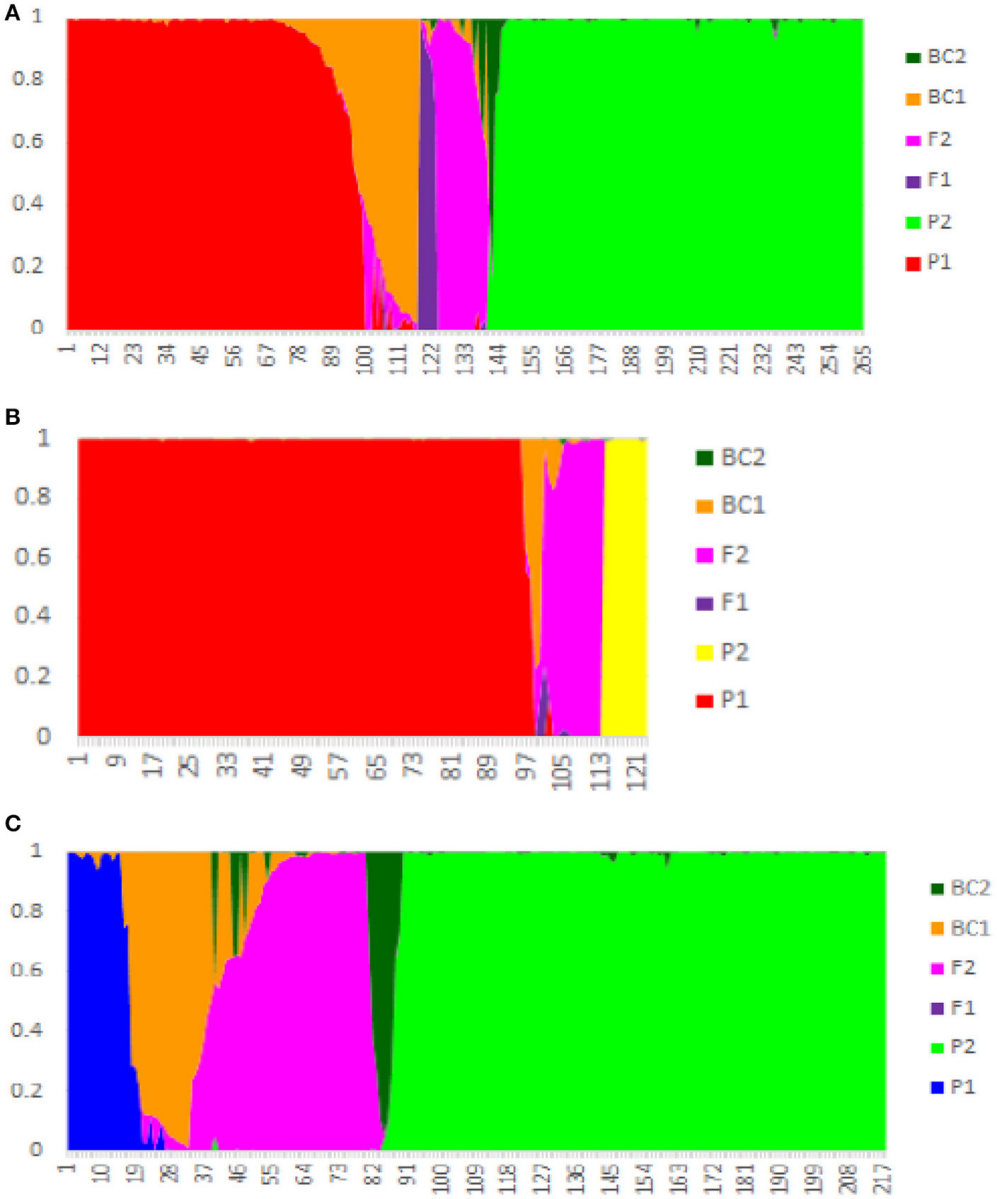
NewHybrid analysis based on nSSR data of 402 tea samples from China and India. **(A)** Chinese Assam type tea as P1 and China type tea in China as P2; **(B)** Chinese Assam type tea as P1 and *Camellia taliensis* as P2; **(C)** Indian Assam type tea as P1 and China type tea from China and India as P2.

Among the 119 hybrid samples included here, only six (5.0%) were identified as F_1_ hybrids, while 64 (53.8%) were assigned as F_2_ hybrids, and 30 (25.21%) as backcross (BC) hybrids. The remaining 16 samples showed a complex genetic admixture from at least three tea group gene pools, suggesting that they underwent a complex breeding process and were excluded from the NewHybrids analysis (Figure 3 and Table 2). Among the 30 CICM samples (mainly from Darjeeling, India), 22 were F_2_ hybrids between Indian Assam type tea and China type tea and five were backcrosses to Indian Assam type tea. The remaining three samples (144, 190, and 192) were assigned as China type tea, although they were defined as hybrids in the STRUCTURE analysis. Of the 22 samples of ACLM, 11 were defined as hybrids (10 F_2_ and one BC_2_) between Chinese Assam type tea and *C. taliensis* and seven represented complex hybrids involving more than two parents. The remaining four samples were identified as representing BC_1_ (3 hybrids) and one F_1_ hybrid between Chinese Assam type tea and China type tea. Among the 45 ACCM samples, 33 were assigned as hybrids between Chinese Assam type tea and China type tea, six were hybrids between Chinese Assam type tea and *C*. *taliensis* (three F_2_ and three BC_1_) and six showed multiple hybridization events with more than two possible parents. All 10 samples of AICM were F_2_ hybrids between Indian Assam type tea and China type tea (Table 2). In addition, a total of 42 samples that were identified as “pure” in STRUCTURE were assigned as hybrids according to NewHybrids analysis (Table S2). Out of these, 31 were Indian Assam type tea, which included 22 BC_1_ and nine F_2_ hybrids, the remaining 11 samples (CCC: 4; ACL: 2 and ACC: 5) were BC hybrids.

**Table 2:** Parentage and types of hybrids in the Mosaic group obtained using NewHybrids.

**Mosaic category**	**P1 (maternal)**	**P2 (paternal)**	**F_1_**	**F_2_**	**BC1**	**BC2**	**Total**
CCLM	Chinese Assam type tea	China type tea				1	1^*^
CCCM	Chinese Assam type tea	China type tea	1	3			4
	Indian Assam type tea	China type tea		1		3	4
	Complex^***^						3
CICM	Indian Assam type tea	China type tea		22	3	2	27
		China type tea					3^**^
ACLM	Chinese Assam type tea	*C. taliensis*		10		1	11
	Chinese Assam type tea	China type tea	1		3		4
	Complex^***^						7
ACCM	Chinese Assam type tea	*C. taliensis*		3	3		6
	Chinese Assam type tea	China type tea	4	15	14		33
	Complex^***^						6
AICM	Indian Assam type tea	China type tea		10			10
Total			6	64	23	7	119

* Single sample of China type tea landrace was belonging to Mosaic group, hence a separate CCLM category was not formed;

** Three samples of CICM were assigned to Parental China type tea in NewHybrid analysis;

*** *Complex: 16 samples in total showing a complex mixing of more than two parental genepools according to STRUCTURE results were defines as “Complex” hybrids and excluded from analysis*.

### cpDNA sequence data and genetic relationships

The length of the three cpDNA regions, *ndh*F*-rpl*32, *trn*SGG*-trn*Sr, *trn*Sf1*-trn*GGG was 662, 692, and 581 bp, respectively. A total of 31 haplotypes were defined for the 101 samples that were sequenced (Table S3). Four haplotypes were defined for five *C. taliensis* samples, of which H6 (TMK2) and H7 (TBW1) were private, while H15 (TY7, TWQ1) and H17 (TXW1) were shared with five landrace samples of Chinese Assam type tea (Table S3). H1 was the dominant haplotype of China type tea detected in the 22 samples that were from China and India, as well as in the hybrids of China type tea (MW153, 144) and a Chinese Assam type tea sample (704). H22 and H24 were the two dominant haplotypes of Indian Assam type tea, but were also present in a few samples of China type tea hybrids from India. H11 and H23 were two haplotypes that were private to Indian Assam type tea. Chinese Assam type tea had a dominant haplotype H26 and eight private haplotypes (H10, H12, H14, H16, H21, H29, H30, and H31). For the 101 sequenced samples, 43 were found to be hybrids according to the NewHybrids and STRUCTURE analyses (Table S3).

**Figure 4:**
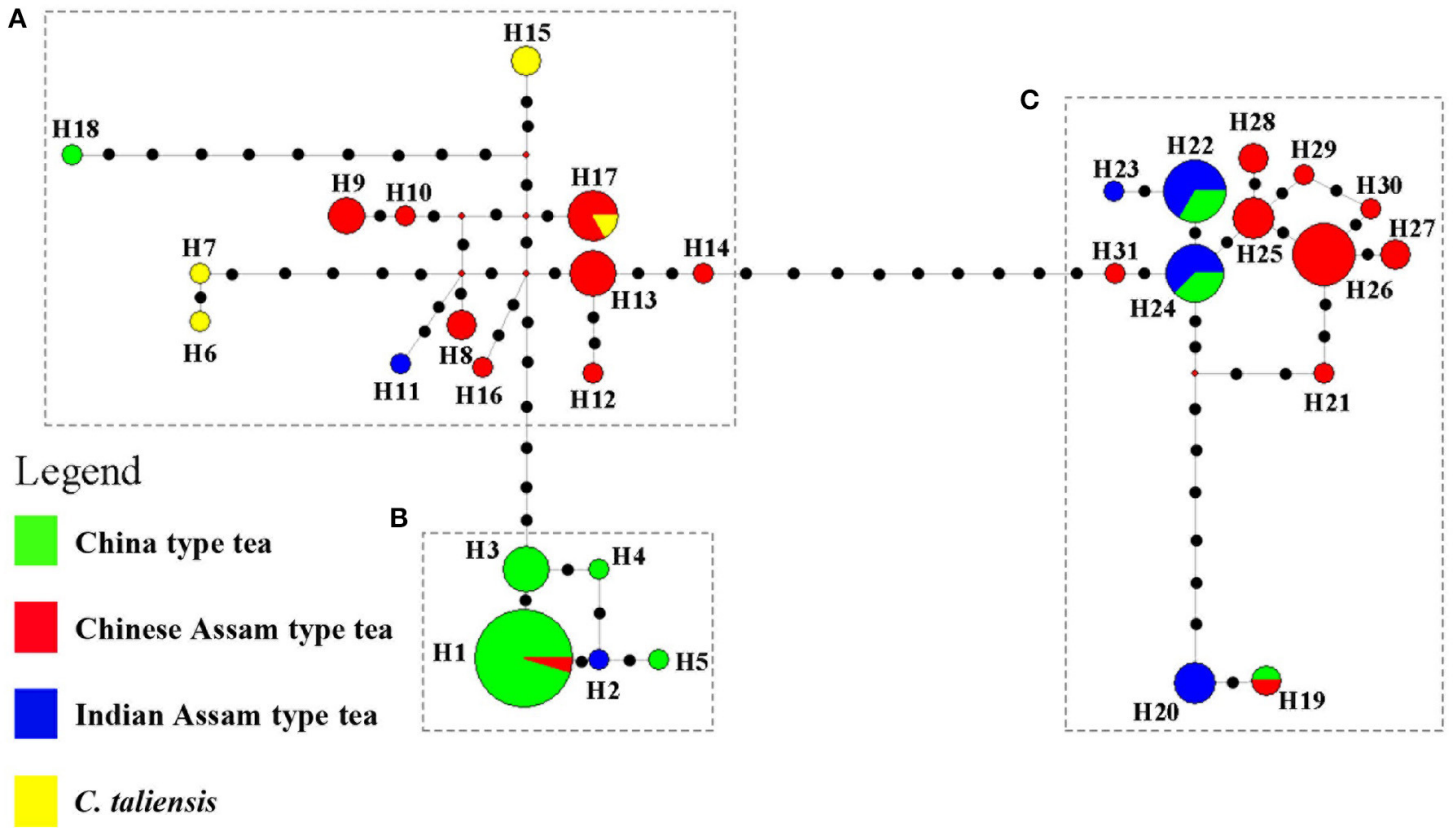
NETWORK tree for 31 cpDNA haplotypes of 101 tea samples from China and India. Circle sizes in the Network are proportional to the haplotype frequencies. Black dots on the lines shows mutation points. Clade **A** Haplotypes of Chinese Assam type tea from Southern Yunnan and *Camellia taliensis*; Clade **B** Haplotypes predominantly of China type tea; Clade **C** Haplotypes of Indian Assam type tea and Chinese Assam type tea from Western Yunnan.

The network tree for the 31 haplotypes was separated into three major clades (Figure 4). Clade A included thirteen haplotypes: three private haplotypes of *C. taliensis*, eight haplotypes of Chinese Assam type tea from Southern Yunnan (Xishuangbanna and Pu'er) of China, and one private haplotype of Indian Assam type tea (H11). This clade also included one private haplotype (H18) of China type tea which showed a distinct divergence from the other China type tea samples in Clade B. Clade B comprised four haplotypes of China type tea (H1, H3, H4, and H5) plus one haplotype (H2) private to Indian Assam type tea. Clade C contained eight haplotypes of Chinese Assam type tea that were mainly from Western Yunnan (Lincang and Baoshan) in China as well as four haplotypes of Indian Assam tea. No haplotype was shared between Chinese Assam type tea and Indian Assam type tea.

**Figure 5:**
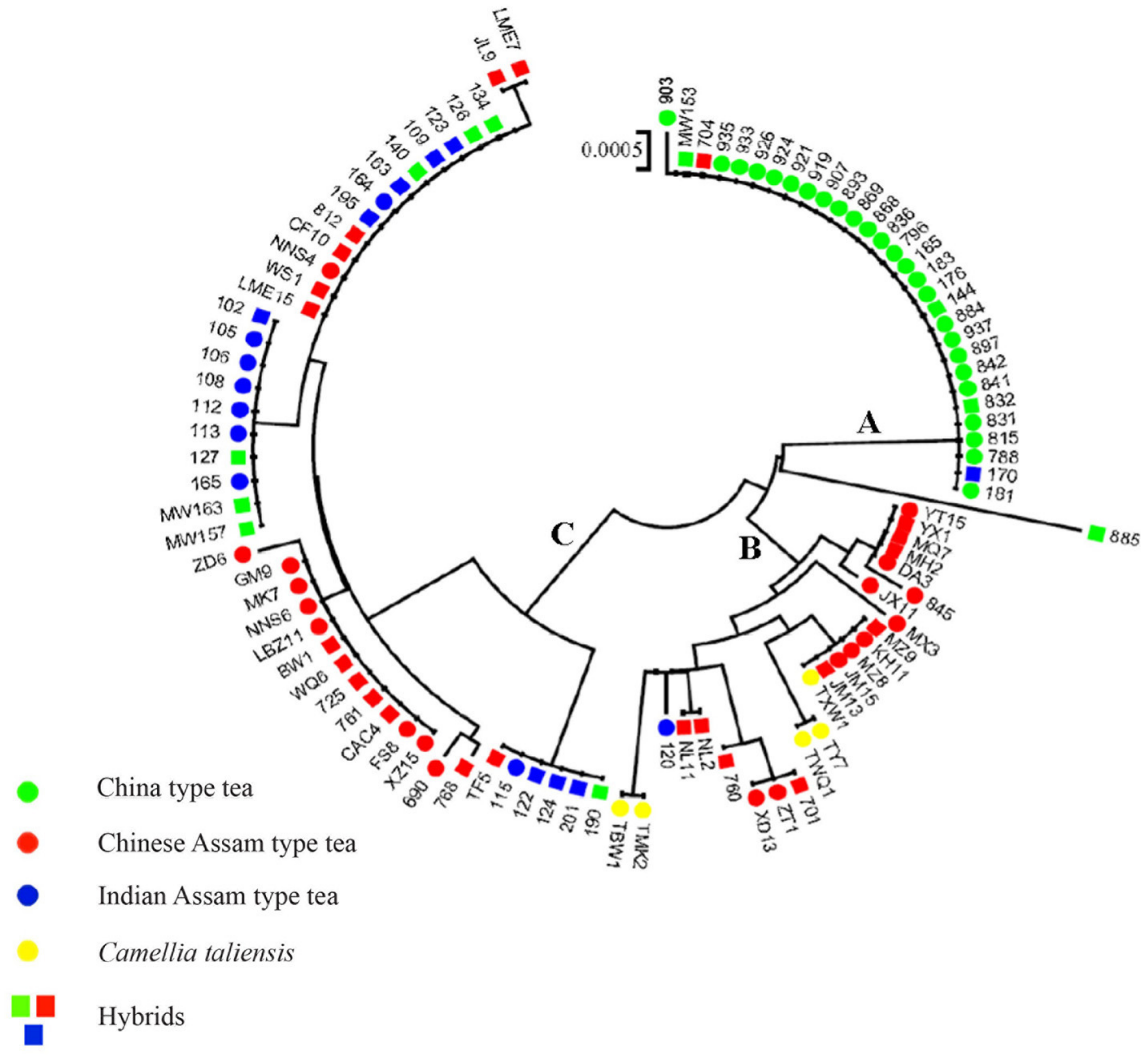
The NJ tree of 101 tea samples from China and India based on cpDNA sequence data. Clade A, haplotypes predominantly of China type tea; Clade B, haplotypes of Chinese Assam type tea mostly from Southern Yunnan and *Camellia taliensis*; Clade C, haplotypes of Indian Assam type tea and Chinese Assam type tea from Western Yunnan. 43 Samples which were shown to be hybrids based on nSSR data with STRUCTURE and a NewHybrids analysis are shown with squares with the colors referring to the morphological characteristics.

The NJ tree of the 101 sequenced samples showed similar relationships to those in the haplotype network tree (Figure 5). Clade A consisted of all haplotypes of China type tea and also included several hybrid samples of China type tea (832, MW 153, 144) (Table S2). The China type tea sample 885 collected from Jiangxi was sister to the remaining samples of this clade. Clade B included haplotypes of Chinese Assam type tea from South Yunnan, *C. taliensis*, and the haplotype of one Indian Assam type tea sample (120). Some hybrid Chinese Assam type tea samples (YX 1, MQ 7, MH 2, MZ 9, JM 13, NL 2, NL 11, 701, and 760) were also in this clade. Cluster C comprised haplotypes of Chinese Assam type tea from Western Yunnan, Indian Assam type tea and hybrids of China type tea from India.

## Discussion

### New insights into the origin and domestication of the tea plant

In a previous study, we defined three distinct genetic lineages among tea plant accessions; Southern China, the Southwestern Yunnan Province of China, and Assam of India, representing the three potential areas of domestication of China type tea, Chinese Assam type tea and Indian Assam type tea, respectively (Meegahakumbura et al., 2016). In the present study, the existence of three independent gene pools and separate origins for the tea plant was confirmed by the cpDNA data. Chinese Assam type tea, however, which formed a distinct genetic group based on nSSR data (Figure 2), had cpDNA haplotypes that grouped into two distinct clades in the network (Figure 4) and NJ trees (Figure 5). The haplotypes of Chinese Assam type tea from Southern Yunnan (Pu'er and Xishuangbanna) of China formed a distinct clade with haplotypes of *C. taliensis*, whereas the haplotypes of Chinese Assam type tea from Western Yunnan (Lincang and Baoshan) grouped together with haplotypes of Indian Assam type tea. We speculate that the Southern Yunnan Chinese Assam type tea possibly had a different maternal parent than the Western Yunnan Chinese Assam type tea. This pattern may be indicative of two possible independent origins for Assam type tea in Yunnan, China. The inconsistency between nSSR and cpDNA results for the Chinese Assam type tea may be explained by historical exchanges of tea germplasm (Freeman and Ahmed, 2010) and frequent gene flow (especially in the modern tea cultivar breeding programs) between the two neighboring regions in Yunnan which, in turn, might have resulted in the homogenization of the nuclear genetic composition and has obscured pre-existing genetic boundaries. Although the cpDNA haplotypes of Chinese Assam type tea in Western Yunnan were genetically close to Indian Assam type tea, with an exception of the hybrid TF 5 with haplotype H19 (Figure 4), no haplotype was shared between each other. This result agreed with our previous results and confirms the existence of independent domestication events of China type and Indian Assam type teas (Meegahakumbura et al., 2016).

Our results indicated that Chinese Assam type tea in Western Yunnan and Indian Assam type tea in Assam of India may have arisen from a single ancestral population from an area where Southwest China, Indo-Burma, and Tibet meet (Kingdon-Ward, 1950). From this origin of domestication, consecutive independent domestication events likely occurred in Western Yunnan of China and Assam of India. In addition, both nSSR and cpDNA results indicated that China type tea is a distinct genetic lineage, which suggests a separate domestication event for this tea type (Meegahakumbura et al., 2016). Although we propose Southern China as the area of domestication of China type tea, the specific origin of domestication remains unknown due to the lack of wild populations. Thus, future studies that include a broader sampling of wild relatives of the tea plant as well as more samples of ancient trees from or near wild populations would likely enhance our resolution of the exact origin of domestication of tea in China.

### Demographic modeling and the beginning of tea domestication

The tea plant was initially domesticated in China over 4,000 year ago (Yamanishi, 1995). Demographic modeling with Approximate Bayesian Computing (ABC) has been used to estimate divergence times of crop plants based on the evolutionary rates of nSSR data (Carnille et al., 2012; Diez et al., 2015). Furthermore, Duke (1983) proposed a generation replacement time (from seed to seed) in *Camellia sinensis* of between 4 and 12 years. Given a generation time of 12 years during pre-cultivation where extensive fertilizer applications and other high-tech practices were not applied, we calculated China type tea and Assam type tea as having first diverged approximately 22,000 years ago, and Chinese Assam type tea and Indian Assam type tea as having diverged from each other approximately 2,770 years ago. These results further strengthen the hypothesis of three genetic lineages of the tea plant as proposed in our previous study (Meegahakumbura et al., 2016). The divergence time between China type tea and Assam type tea roughly coincides with the last glacial maximum (LGM) (Cook et al., 2011, 2012; Yao et al., 2015). During this period, some ancestral populations of China type tea (*C. sinensis* var*. sinensis*) possibly migrated southward from its northern distribution range (such as East and/or Central China) to isolated Southern refugia, possibly in the mountains of South and West Yunnan, and East Himalayas, and then subsequently diverged into the two varieties, *C. sinensis* var. *sinensis* and *C. sinensis* var. *assamica*. At this point in time human interference was unlikely (Endicott et al., 2009). However, the divergence time calculated between Chinese Assam type tea and Indian Assam type tea (2,770 years ago) is consistent with early historical records of tea usage in Yunnan (Zhao and Yin, 2008) and might be indicative of an early tea domestication event in China, much earlier than the documented usage of tea in India (Das et al., 2012).

### Tea breeding history and cultivar improvement in China and India

China has a long history of tea breeding. It is reported that tea cultivars were selected based on morphological traits from as early as the eighth century (Wu, 1987). In the present study, almost a third (119 out of the 402 tea samples analyzed) were categorized as hybrids between tea types or between the tea plant and its wild relative *C. taliensis*, for both landraces and modern cultivars. This result indicates that apart from selection, hybridization (occurring naturally or by artificial pollination) has played an important role in tea domestication. For hybrid samples defined by NewHybrid analysis (Table 2 and Table S2), only six samples were F_1_ hybrids, while most were F_2_ and BC hybrids. These results are similar to the results that have been reported in East Africa (Wambulwa et al., 2016a) where approximately one third of the tea samples (64 out of 193 samples) were found to be F_2_ and BC hybrids. It also seems likely that closely related wild relatives of the tea plant have contributed to tea cultivar development in China (Li et al., 2015). For example, the Indian Assam type tea samples 115, 122, 124, 201, possess haplotype H20, which is genetically distinct from other Indian Assam type tea cultivars (Figure 5). Furthermore, results of BLAST searches in GenBank for the three cpDNA regions used in this study matched the wild species *C. pubicosta*. These samples are likely F_2_ hybrids or backcross generations between Indian Assam type tea and *C. pubicosta* as the maternal parent suggesting the introduction of wild tea plant germplasm into the Indian tea breeding program since at least before 1890, the planting date of one of our samples (201). Similarly, sample 885, which is a backcross hybrid (Table S2) possessing the H18 haplotype, possibly hybridized with a wild *Camellia* species of *Camellia* sect. *Thea*. Collectively, our results indicate that germplasm of wild species of *Camellia* was involved in the domestication and breeding of the tea plant.

Comparison of the breeding histories in different tea domestication regions could provide valuable information for the direction of future breeding programs. Unfortunately, different countries have adopted different breeding objectives and have applied different genetic systems (markers) to identify germplasm and confirm lineages, which makes it difficult to make comparisons among studies (Chen et al., 2012). This study is the first to use the same genetic markers to compare the origin and breeding history of the three main tea types currently in cultivation in China and India. Our results indicated that China type tea, Chinese Assam type tea and Indian Assam type tea may have undergone different breeding processes in their respective domestication regions. More specifically, China type tea was possibly derived from a single domestication event in Southern China (Meegahakumbura et al., 2016). This suggests that the largely “pure” genetic composition of China type tea might be attributable to the limited gene flow between China type tea and its wild relatives, the wild species is not in the close vicinity of major growing areas of China type tea in China. Interestingly, the landraces of China type tea showed a lower proportion of hybrids compared to modern China type tea cultivars (2.7 vs. 15%), but exhibited relatively higher gene diversity than the cultivars (*He* = 0.692 vs. *Hs* = 0.680). This result might indicate that the seeds of China type landraces were collected from a relatively wide gene pool, while modern cultivars may have been bred by selection and/or artificial hybridization involving a few tea individuals representing a limited gene pool (Tan et al., 2015). Two modern cultivars of China type tea collected from Guangdong, China are shown to be hybrids with Indian Assam type tea (MW157, MW163), indicating that Indian Assam tea has been used recently in tea breeding programs in China. They both possess a maternal lineage of Indian Assam type tea (Figure 5), thus China type tea was used as paternal parent. Overall, our results indicated that hybridization between the tea types and their closely related wild species, and/or among different tea types might have played a major role in tea breeding.

The cpDNA results revealed that Chinese Assam type tea possibly has two origins in China (Southern and Western Yunnan) and possesses high haplotype diversity. According to the nSSR analysis, landraces of Chinese Assam type tea exhibited relatively higher gene diversity than cultivars (*He*: 0.633 vs. 0.618). Natural introgression from other sympatric wild relatives in this region could have possibly contributed to the higher gene diversities found in this study. For example, approximately 50% of landrace samples of Chinese Assam type tea (ACLM) exhibited a genetic admixture between Chinese Assam type tea and *C. taliensis* (Figure 2 and Table 2). Furthermore, *C. taliensis* haplotype H17 was shared with several samples of Chinese Assam type tea (Figure 4; Table S3). *C. taliensis* is a related wild species of the tea plant that has a long history of cultivation in ancient tea gardens alongside Chinese Assam type tea plants in Yunnan (Zhao et al., 2014). Thus, gene flow between Chinese Assam type tea and *C. taliensis* has likely resulted in Chinese Assam type tea having relatively higher genetic diversity in hybrid individuals. In turn, it seems likely hybrid landraces were selected by farmers for their unique taste, due to their low caffeine and high theobromine content (Ogino et al., 2009). Interestingly, more than 73% (33 out of 45) of the samples of ACCM (Figure 2; Table 2) showed genetic admixture between China type tea and Chinese Assam type tea, with the latter usually serving as the maternal parent. In addition, 13 hybrid landraces / cultivars of Chinese Assam type tea showed genetic admixture from more than two species/tea types. This suggested that multiple hybridization events between landraces/cultivars and wild species/different tea types may have occurred during the breeding of tea cultivars. Collectively, these results indicated that modern breeding programs should implement mating between distinct tea types or related wild species via artificial hybridization to breed new cultivars and bolster genetic diversity of germplasm stocks.

Although the history of tea plantations in India is relatively short, less than 200 years (Das et al., 2012), the Indian Assam type tea has been proposed to have been domesticated independently in Assam of India (Meegahakumbura et al., 2016). It is known that China type tea germplasm was introduced to India in 1836 (Sharma et al., 2010). However, it did not grow well and was hybridized with indigenous tea accessions and wild relatives from a wide range of geographic areas in India. Subsequently, the selection of tea cultivars was performed from a highly heterogeneous gene pool (Sharma et al., 2010). The high genetic diversity and higher proportion of F_2_ and BC hybrids among the accessions of Indian Assam type tea detected in our study (Table 2; Table S2) possibly reflects early hybridization events between the introduced China type tea and local Assam tea accessions, and maybe even with wild species of *Camellia* in India (Raina et al., 2011). This seems likely given that all the commercial cultivars in our study that were from Darjeeling, India were found to be hybrids between China type tea and Indian Assam type tea, and most of them with Indian Assam type tea serving as the maternal parent. Furthermore, China type tea, which naturally comes from cold climates, might have contributed cold tolerance genes to hybrids typically found in high elevations of the Darjeeling area. Thus, hybrids between different tea types exhibiting higher environmental adaptability or possessing more favored agricultural traits than their parents, may have played a substantial role in tea cultivation in India and China.

### Implications for tea germplasm conservation

The three distinct types of the tea plant represent a rich gene pool, which is valuable for future tea breeding and cultivar development. For example, Assam tea from India and China type tea are the only two gene pools that are currently used for the development of hybrid tea cultivars in approximately 30 countries worldwide (Ellis, 1995). In particular, Chinese Assam type tea from Southwestern Yunnan, China was recently found to represent a distinct gene pool (Meegahakumbura et al., 2016) and both landraces and cultivars of Chinese Assam type tea possessed a high proportion of rare alleles and private haplotypes. Given these results, Chinese Assam type tea is a highly valuable, yet underutilized, germplasm resource for future tea breeding. Furthermore, the existence of ancient trees that are presumed to be over 1,000 years old may present a unique genetic window into tea domestication in China. Therefore, the preservation of these tea plant germplasm resources through *in-situ* conservation should be considered a conservation priority in Yunnan, China.

## Data accessibility

The Microsatellite Data sets supporting the results in this study have been deposited in the Dryad Digital Repository under the doi: 10.5061/dryad.6gq1g. The cpDNA sequence data in this study have been deposited in NCBI GenBank under accession numbers MG676240-MG676332 as shown in the Table S3.

## Author contributions

L-MG, D-ZL, and J-CX conceived and designed the study. MKM, M-ML, KT, L-MG, J-BY, B-YL, and J-CX collected plant material. MKM, M-ML and MW performed the experiments. MKM, MW, Y-SS, JL, MM, and L-MG analyzed and interpreted the data. MKM, MW, Y-SS, JL, MM, J-CX, D-ZL, and L-MG wrote and revised the manuscript. All authors reviewed and approved the final manuscript.

### Conflict of interest statement

The authors declare that the research was conducted in the absence of any commercial or financial relationships that could be construed as a potential conflict of interest.

## Supplementary material

Figure S1 Best *K* values for STRUCTURE analysis using two methods of **(A)** Δ*K* and **(B)** Log Likelihood (*K*).

Table S1 SSR dataset for *Camellia taliensis*.

Table S2 Collection Information of the 402 tea samples used in the current study.

Table S3 Details of the haplotypes obtained with cpDNA sequencing.
