# Supplementary material for: Domestication Origin and Breeding History of the Tea Plant (Camellia sinensis) in China and India Based on Nuclear Microsatellites and cpDNA Sequence Data
Source: Front Plant Sci. 2018 Jan 25;8:2270. doi: 10.3389/fpls.2017.02270 (PMC5788969; doi:10.3389/fpls.2017.02270)
Supplement: Figure S1 — Best K values for STRUCTURE analysis using two methods of (A) ΔK and (B) Log Likelihood (K). [file Image1.pdf]

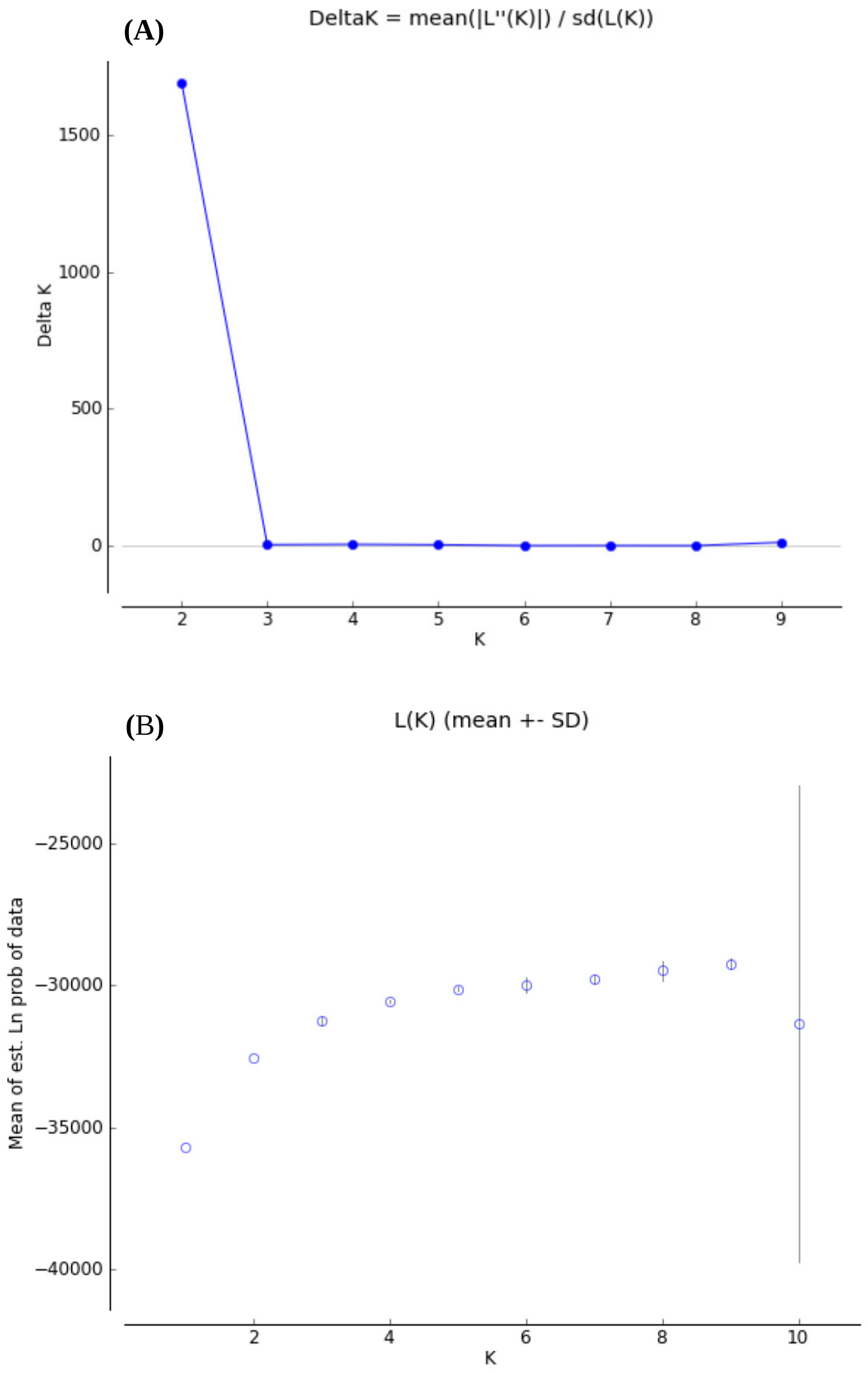

Figure S1 Best *K* values for STRUCTURE analysis using two methods of (A) *ΔK* and (B) Log Likelihood (*K*).
