# Supplementary material for: Domestication Origin and Breeding History of the Tea Plant (Camellia sinensis) in China and India Based on Nuclear Microsatellites and cpDNA Sequence Data
Source: Front Plant Sci. 2018 Jan 25;8:2270. doi: 10.3389/fpls.2017.02270 (PMC5788969; doi:10.3389/fpls.2017.02270)
Supplement: Table S3 — Details of the haplotypes obtained with cpDNA sequencing. [file Table3.DOC]

**Table S3: Details of the haplotypes obtained with cpDNA** sequencing

| **Haplotype** | **Sample IDs** | **Number** | **GenBank accession number**  **ndhF-rpl32, trnSf1-trnGGG, trnSGG-trnSr** |  | |
| --- | --- | --- | --- | --- | --- |
| H1 | 181, 183, **704**, 788, 796, 815, 831, **832**, 836, 841, 842, 868, | 22 | MG676240, MG676271, MG676302 |  |  |
|  | 869, 884, 893, 897, 924, 926, 933, 935, 937, **MW153** |  |  |  |  |
| H2 | **170** | 1 | MG676241, MG676272, MG676303 |  |  |
| H3 | **144**, 185, 907, 919, 921 | 5 | MG676242, MG676273, MG676304 |  |  |
| H4 | 176 | 1 | MG676243, MG676274, MG676305 |  |  |
| H5 | 903 | 1 | MG676244, MG676275, MG676306 |  |  |
| H6 | TMK2 | 1 | MG676245, MG676276, MG676307 |  |  |
| H7 | TBW1 | 1 | MG676246, MG676277, MG676308 |  |  |
| H8 | **NL2**, **NL11** | 2 | MG676247, MG676278, MG676309 |  |  |
| H9 | XD13, **701**, ZT1 | 3 | MG676248, MG676279, MG676310 |  |  |
| H10 | **760** | 1 | MG676249, MG676280, MG676311 |  |  |
| H11 | 120 | 1 | MG676250, MG676281, MG676312 |  |  |
| H12 | 845 | 1 | MG676251, MG676282, MG676313 |  |  |
| H13 | DA3, **MH2, MQ7**, YT15, **YX1** | 5 | MG676252, MG676283, MG676314 |  |  |
| H14 | JX11 | 1 | MG676253, MG676284, MG676315 |  |  |
| H15 | TY7, TWQ1 | 2 | MG676254, MG676285, MG676316 |  |  |
| H16 | MX3 | 1 | MG676255, MG676286, MG676317 |  |  |
| H17 | TXW1, **JM13**, JM15, MZ8, **MZ9**, KH11 | 6 | MG676256, MG676287, MG676318 |  |  |
| H18 | **885** | 1 | MG676257, MG676288, MG676319 |  |  |
| H19 | **TF5**, **190** | 2 | MG676258, MG676289, MG676320 |  |  |
| H20 | 115, **122**, **124**, **201** | 4 | MG676259, MG676290, MG676321 |  |  |
| H21 | ZD6 | 1 | MG676260, MG676291, MG676322 |  |  |
| H22 | **102**, 105, 106, 112, 113, **127**, 165, **MW157**, **MW163** | 9 | MG676261, MG676292, MG676323 |  |  |
| H23 | 108 | 1 | MG676262, MG676293, MG676324 |  |  |
| H24 | **109**, **123**, **126**, **134**, **140**, **163**, 164, **195** | 8 | MG676263, MG676294, MG676325 |  |  |
| H25 | **812**, **CF10**, NNS4, **WS1** | 4 | MG676264, MG676295, MG676326 |  |  |
| H26 | **725**, **761**, GM9, NNS6, LBZ11, **BW1**, **CAC4**, XZ15, FS8 | 9 | MG676265, MG676296, MG676327 |  |  |
| H27 | MK7, **WQ6** | 2 | MG676266, MG676297, MG676328 |  |  |
| H28 | **JL9**, **LME7** | 2 | MG676267, MG676298, MG676329 |  |  |
| H29 | **768** | 1 | MG676268, MG676299, MG676330 |  |  |
| H30 | 690 | 1 | MG676269, MG676300, MG676331 |  |  |
| H31 | **LME15** | 1 | MG676270, MG676301, MG676332 |  |  |
| **Total** |  | **101** |  |  |  |

Note: 43 Mosaic/hybrid samples with STRUCTURE/NewHybrid analysis based on SSRs results used for cpDNA sequencing are marked in **bold**. Samples underlined are the 31 samples of which cpDNA sequences were submitted to the GenBank.
